# Improved response inhibition induced by attentional capture is associated with physical activity

**DOI:** 10.7717/peerj.14083

**Published:** 2022-09-26

**Authors:** Hao Zhu, Jiuyang Xu, Yue Zheng, Guiping Jiang, Xinyi Huang, Xiaohuan Tan, Xueping Wu

**Affiliations:** 1 School of Psychology, Shanghai University of Sport, Shanghai, China; 2 Tongda College, Nanjing University of Posts and Telecommunications, Yangzhou, China; 3 School of Physical Education, Jiangsu Vocational and Technical College of Economics and Trade, Nanjing, China; 4 School of Physical Education and Training, Shanghai University of Sport, Shanghai, Shanghai, China; 5 School of Physical Education, Harbin University, Harbin, China

**Keywords:** Physical activity, Response inhibition, Attentional capture, Stop signal task, Inhibitory control

## Abstract

The ability to stop a response promptly when a stop signal is presented is named response inhibition. It is generally accepted that the process of response inhibition requires a subject to pay attention to the stop instruction and then cancel the action. A wealth of converging evidence suggests that physical activity (PA) can promote response inhibition, but the potential contributions of attentional capture to the relationship between PA and response inhibition are currently unknown. In this study, the standard stop-signal task (SST) and two novel versions of the SST were used to solve this gap. A total of 58 college students were divided into a higher PA group and a lower PA group, respectively. In Experiment 1, the classical SST determined that the participants in the higher PA group displayed a significantly faster stop-signal reaction time (SSRT) than those in the lower PA group. Experiment 2 separated the attentional capture in the SST and revealed that the participants in the higher PA group could detect the signal faster than those in the lower PA group. Experiment 3 further added a stop signal to Experiment 2 and demonstrated that the participants in the higher PA group could more effectively deploy attentional resources to complete the task. Overall, these findings indicate that PA is positively associated with response inhibition and that the positive relationship is associated with effective allocation of attentional resources for faster attentional capture.

## Introduction

Physical activity (PA) has been shown to be associated with many cognitive functions (Erickson et al., 2019; Vazou et al., 2019), including inhibitory control (Aly & Kojima, 2020; Haverkamp et al., 2020; Zhu et al., 2021). Inhibitory control is a complex executive function that can be subdivided into motor response inhibition and interference inhibition (Wöstmann et al., 2013). Motor response inhibition refers to the ability to stop a response promptly when a stop signal is presented (Chambers, Garavan & Bellgrove, 2009; Mirabella, 2021) while interference inhibition is a more cognitive form of inhibitory control which measures the ability to resolve response conflict due to irrelevant but incompatible stimulus features (Mirabella, 2021). The former has been assumed to be more likely to be directly affected by PA (Rey-Mermet & Gade, 2018).

The stop-signal task (SST) has been widely used to investigate response inhibition as it provides a measure of the time required to complete inhibitory processes, *i.e*., the stop-signal reaction time (SSRT) (Logan, Cowan & Davis, 1984). It is worth noting that the SST requires high cognitive demands, involving both response inhibition and attentional capture (Sharp et al., 2010; Lee et al., 2016; Xu et al., 2017; Wang et al., 2019). The unexpected onset of a salient signal can result in a delayed reaction time (RT) even when the signal is irrelevant, an effect known as attentional capture (Yantis, 1993). In other words, successful implementation of inhibitory control requires individuals to quickly and effectively detect the appearance of a signal, appreciate its significance, and then to make the correct response. Nevertheless, the standard SST cannot provide an indicator to directly measure attentional capture. Therefore, previous studies have determined that PA can promote response inhibition through the positive relationship between PA and SSRT, which seems to obscure the role of attentional capture (Padilla et al., 2013; Padilla, Pérez & Andrés, 2014). By far, it is unclear whether this association reflects the direct impact of PA on response inhibition or whether the effect is mediated *via* attentional capture.

Although the mechanism by which PA affects response inhibition is not fully understood, recent seminal studies provide strong evidence that the brain regions involved in response inhibition are not the same as those involved in attention (Chikazoe et al., 2009; Dodds, Morein-Zamir & Robbins, 2011; Lee et al., 2016) and the dissociation of the two components was proved by several studies, specifically that right inferior frontal gyrus (rIFG) is primarily associated with attentional capture of task-related signals (Schaum et al., 2021) and that pre-supplementary motor area (pre-SMA) is directly responsible for response inhibition (Wolpe et al., 2022). In addition, attentional capture of unexpected stimuli can optimize subsequent response outputs during the occurrence of goal behaviors (Tang & Chen, 2013; Jiang et al., 2014). These results suggest that attentional capture is one of the determinants of response inhibition. A current systematic review has demonstrated that individuals with a greater participation in PA can effectively allocate limited attentional resources during cognitively demanding tasks (de Sousa et al., 2019). Furthermore, the latest research provided promising evidence that PA can improve the inhibitory control of attentional networks in young adults through attentional network test (ANT). It is worth noting that the Flanker task (a paradigm used to measure interference inhibition) was adopted to evaluate inhibitory control (Meng et al., 2022). Therefore, the field of sports psychology needs a relevant and timely topic to reveal how physical activity influences response inhibition and sustained attention.

The present study aimed to determine whether the association between PA and response inhibition stems from the fact that PA accelerates attentional capture of conflict signals, and further examined whether PA can regulate the allocation of attentional resources to better complete cognitively demanding task. To address this issue, the standard SST and two novel versions of the SST adapted from Erika-Florence, Leech & Hampshire (2014) were used to identify the potential contribution of attentional capture underlying the association between PA and response inhibition. In Experiment 1, the standard SST was administered to investigate the association between PA and response inhibition. In Experiment 2, the rules of the SST were changed, *i.e*., stop trials had a higher probability of being presented than go trials, thus the subjects had a tendency to inhibit the response. In addition, in the go trials, the individual needed to monitor for the go signal. The processing procedure was the same as that of the stop signal in the SST, that is, the reaction tendency conflicted with the low-probability signal. Different from the SST, in Experiment 2, the subjects monitored the appearance of go signal and immediately initiated the key press, and the RT could reflect the subjects’ attentional capture of the signal. Therefore, Experiment 2 can dissociate the impact of PA on attentional capture from its impact on response inhibition. Experiment 3 added a stop signal to Experiment 2, *i.e*., the individual not only needed to inhibit the signal but also was required to respond to the signal. Compared with Experiment 2, the stop signal in Experiment 3 would occupy the attentional resources of the go signal, and the subjects’ attentional resources would be gradually lost with the progression of the experiment. This loss would become more obvious in cognitively demanding tasks.

Based on the results of Experiments 1 and 2, we hypothesized that the participants with higher PA would have a better performance in response inhibition and attentional capture. Furthermore, in Experiment 3, it seems reasonable to suppose that with the progression of the experiment, even if the stop signal would occupy the attention resources of go signal, individuals with higher PA would more effectively allocate limited attentional resources to complete the response to the go signal. By administering these three experiments, we hypothesized that the participants with higher PA would have a better performance in response inhibition and that the key mechanism involves better allocation of attentional resources to enhance attentional capture.

## Materials and Methods

### Participants

A total of 58 college students aged between 20 and 27 years old were recruited for the study. All the participants gave written informed consent prior to participation and met the following criteria: (1) right-handedness, (2) normal vision without colour blindness, (3) reported to be free of psychiatric and neurological disorders. The International Physical Activity Questionnaire—Short Form (IPAQ-SF) was used to measure the total amount of physical activity and to classify the participants into a higher physical activity group (HG) and a lower physical activity group (LG). Participants with gross metabolic equivalents (METs) greater than 3,000 MET-minutes/week were assigned to the HG and those with gross METs equal to or less than 3,000 MET-minutes/week were assigned to the LG. The demographic data of the two groups are presented in Table 1. The study protocol was approved by the Ethics Committee of Shanghai University of Sport (102772020RT054) and was performed in accordance with the Declaration of Helsinki’s guidelines.

**Table 1:** Participant demographics (mean ± standard deviation).

Variable	HG	LG	*p*
N	28	30	
age	23.89 ± 2.02	23.96 ± 1.9	0.89
Gross METs	4,290.27 ± 1,066.78	1,078.13 ± 868.04	<0.001

**Note:**

PA, physical activity; BMI, body mass index; METs, metabolic equivalents; HG, higher physical activity group; LG, lower physical activity group.

### Physical activity measurement

The IPAQ-SF was adopted because it has reliable measurement characteristics for physical activity (Sember et al., 2020; van Tran et al., 2020). The IPAQ-SF contains seven questions regarding the daily cumulative time and frequency of walking, moderate intensity activities, and vigorous intensity activities in the past 7 days. The metabolic equivalent of energy expenditure values for various activities were as follows: 3.3 for walking, 4.0 for moderate intensity activities, and 8.0 for vigorous intensity activities. The final results of the IPAQ-SF are in accordance with the IPAQ scoring protocol guidelines (Ainsworth et al., 2011).

### Tasks

**Figure 1:**
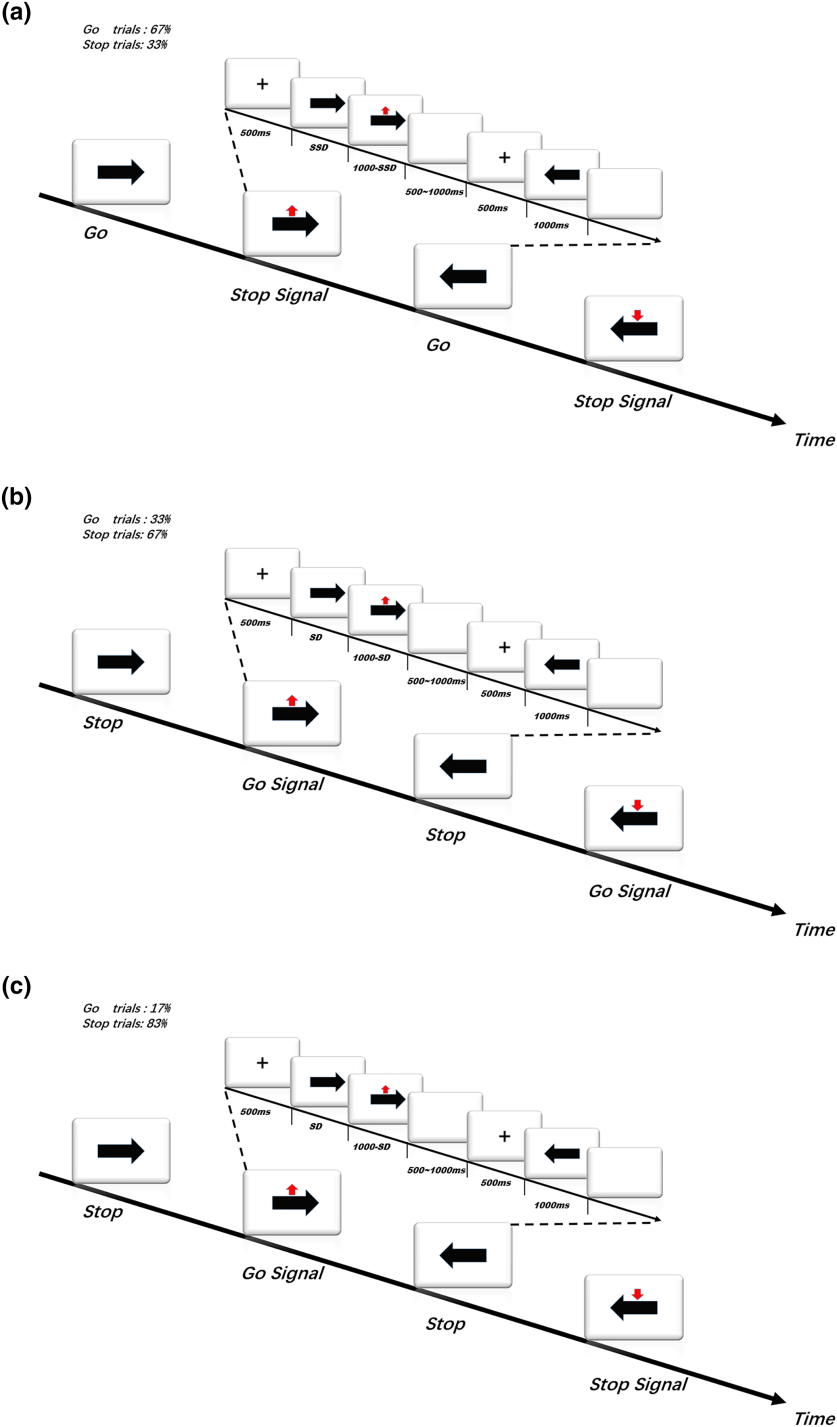
(A) Task in Experiment 1. (B) Task in Experiment 2. (C) Task in Experiment 3.

This study consisted of three experiments. The experimental program was compiled and run by E-Prime 2.0 (Psychology Software Tools, Inc., Pittsburgh, PA, USA). A computer screen (17 inches) was positioned 60 cm in front of the participants. In the formal experiments, all trials were presented in a pseudo-random order, and the red arrow signal appeared no more than twice consecutively. The procedures for each experiment are shown in Fig. 1. First, a “+” with a duration of 500 ms was presented in the centre of the screen, followed by a left or right black arrow, and occasionally an up or down red arrow appeared above the black arrow. The subjects were instructed to press the appropriate key with the index finger of each hand according to the corresponding response rules of each part and to start the next trial after a blank screen appeared for a duration of 500–1,000 ms.

Experiment 1 was a SST. In the frequent go trials (66.6% of trials), a left- or right-pointing black arrow appeared on the screen, and then the subject was instructed to press the “F” key when the black arrow was pointing left and press the “J” key when the black arrow was pointing right quickly and accurately in response to the black arrows. In the infrequent stop trials (33.3% of trials), at a variable delay (stop signal delay, SSD) after the presentation of the go signal, a red arrow pointing upward or downward was presented above the location of the black arrow pointing left or right, and the subject was required to stop pressing the key and not press any additional key. The time interval between the horizontal (go signal) and vertical arrows (stop signal) (*i.e*., SSD) was set according to the staircase tracking algorithm (Rubia et al., 2003). The initial SSD was set to 200 ms, and then it was dynamically adjusted according to the subject’s response: if the subject succeeded at inhibiting the key press in the current stop trial, the SSD of the next stop trial was increased by 34 ms to increase the difficulty of inhibition; if the subject failed at inhibiting the response in the current stop trial, the SSD of the next stop trial would be reduced by 34 ms to facilitate inhibition. This dynamic tracking algorithm allows the SSD change to occur dynamically in a step-by-step manner, ensuring that the subjects can successfully inhibit 50% of the stop trials (Rubia et al., 2001). If the subject failed at inhibiting the key press, the stop signal disappeared after the subject pressed the key. If the subject succeeded at inhibiting the key press, the presentation time of the stop signal was set to (1,000 − SSD) ms. In this study, a relatively more accurate integration method was used to estimate the SSRT. That is, assuming *n* is the probability of the wrong reaction in the stop trial, *t* is the number of correct reactions in the go trial, and the correct Go RT is ordered from fast to slow, SSRT = (*n × t*) th Go RT − mean SSD (Boehler et al., 2012; Verbruggen, Chambers & Logan, 2013).

In Experiment 2, the response rules of the SST were changed to create a conflicting situation. The task required the subjects not to press any key when a black arrow pointing left or right appeared in the frequent stop trials (66.6% of trials). In the infrequent go trials (33.3% of trials), at a variable delay (signal delay, SD) after the presentation of the black arrow, a red arrow pointing upward or downward appeared above the black arrow, and the subjects were instructed to press the “F” key when the black arrow was pointing left and press the “J” key when the black arrow was pointing right quickly and accurately in response to the black arrows. According to previous studies with participants of a similar age distribution (Erika-Florence, Leech & Hampshire, 2014; Hampshire & Sharp, 2015), the value of the SD was set as random, and the range of variation was set as 312 ± 115 ms.

Experiment 3 was based on Experiment 2, but the motor responses on the go trials were more difficult as the subjects had to press the “F” key or the “J” key quickly and accurately only when a red arrow pointing upward appeared above the black arrow (16.7% of trials). On all other trials, no key press was necessary. The variation pattern of the SD in Experiment 3 was the same as that in Experiment 2.

Before each experiment, all subjects were informed about the response rules of the experiment and received 40 practice trials with feedback. After the practice, the subjects were required to orally report whether they had mastered the response rules of the corresponding experiment. If the subjects had not mastered the response rules, they were required to practice again. All subjects completed Experiments 1 to 3 in sequence and were given a 10-min rest between each experiment. In half of the participants (14 in the HG and 15 in the LG), the order in which the tasks were completed was inverted to eliminate order effect. Each experiment contained 180 trials (composed of three blocks, *i.e*., 60 trials for each block), and the red arrow appeared 60 times (red arrows pointing upward appeared 30 times, and red arrows pointing downward appeared 30 times).

### Statistical analyses

All statistical analyses were implemented in SPSS 22.0 (IBM, Armonk, NY, USA). The mean behavioural test values are reported with standard deviations. Independent samples t-tests was used to compare the demographic variables across the two groups. The independent samples t-test was used to compare the SSD, RT, and accuracy across the two groups. Group-by-task repeated-measures analysis of variance (ANOVA) was performed to analyse the difference in RT and accuracy between the two groups under different tasks (Experiments 2 and 3), with follow-up analyses. Group-by-block repeated-measures ANOVA was used to compare the differences in the RT and accuracy between the two groups in the three blocks (Early block, Mid block and Terminal block) of Experiment 3. Multiple tests for the three blocks were validated with Bonferroni correction. A value of *p* < 0.05 indicated a statistically significant difference.

## Results

### Experiment 1

Data are reported in Table 2. The results of the independent samples t-test showed that there was no significant difference in the accuracy of the stop signal trials (Stop accuracy) between the two groups (Stop accuracy ≈ 50%), indicating that the tracking algorithm applied in this experiment is reliable and effective. In addition, the SSRT of the HG was significantly shorter than that of the LG (t_(56)_ = −2.36, *p* < 0.05, Cohen’s d = −0.62). No significant difference was observed in the RT of the go trials (Go RT; t_(56)_ = −1.40, *p* > 0.05, Cohen’s d = −0.37), accuracy of the go trials (Go accuracy; t_(56)_ = 0.96, *p* > 0.05, Cohen’s d = 0.25), or SSD (t_(56)_ = 0.75, *p* > 0.05, Cohen’s d = 0.20) between the two groups.

**Table 2:** Indicators involved in Experiment 1 (mean ± standard deviation).

Variable	HG	LG	*p*
G. RT (ms)	509.01 ± 78.92	538.33 ± 81.00	0.17
G. accuracy	0.98 ± 0.02	0.98 ± 0.03	0.34
Stop accuracy	0.53 ± 0.02	0.52 ± 0.03	0.69
SSD (ms)	298.34 ± 75.41	281.85 ± 91.46	0.46
SSRT (ms)	183.79 ± 86.88	237.53 ± 86.69	0.02

**Note:**

HG, higher physical activity group; LG, lower physical activity group; Go RT, reaction time of the go trials; SSD, stop signal delay; SSRT, stop signal reaction time; PA, physical activity.

### Experiment 2

**Figure 2:**
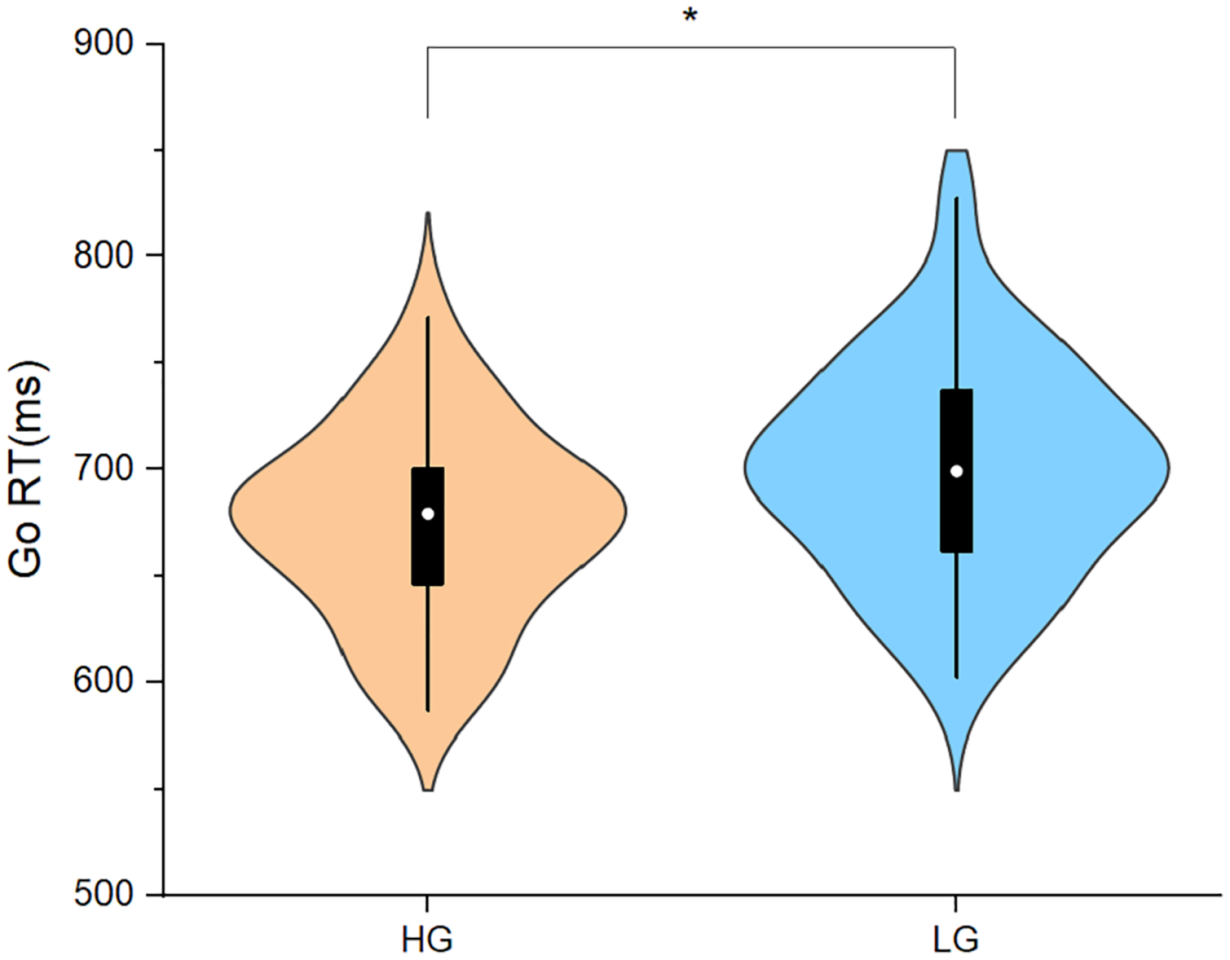
Reaction time of go trials in Experiment 2. Abbreviations: Go RT, reaction time of the go trials; HG, higher physical activity group; LG, lower physical activity group. *p < 0.05.

**Figure 3:**
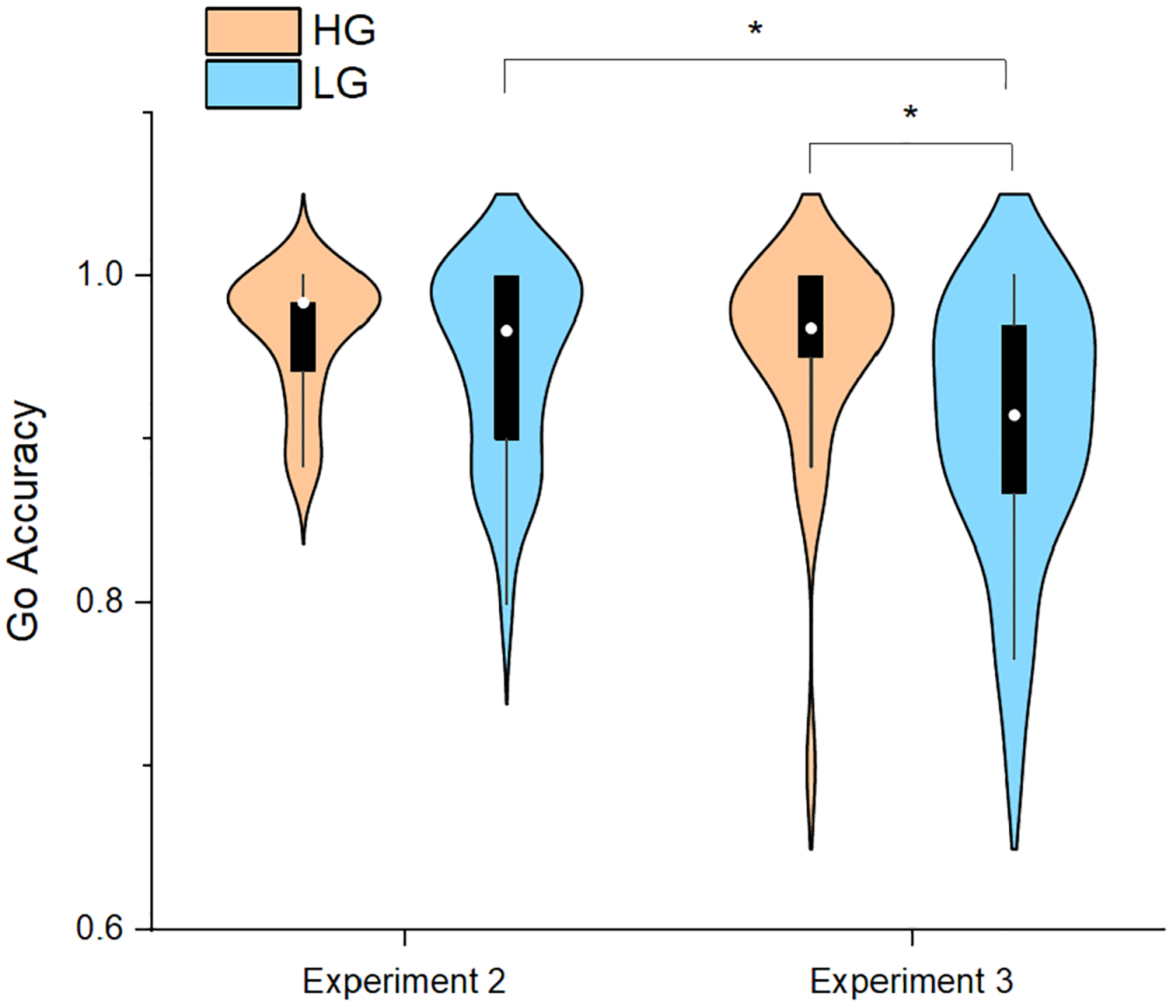
Accuracy of the go trials in Experiment 2 and Experiment 3. Abbreviations: HG, higher physical activity group; LG, lower physical activity group. *p < 0.05.

As shown in Fig. 2. In Experiment 2 the Go RT was significantly shorter in HG than in LG (t_(56)_ = −2.05, *p* < 0.05, Cohen’s d = −0.54). The Go accuracy was not significantly different among the two groups (t_(56)_ = 0.65, *p* > 0.05, Cohen’s d = 0.17), refer to Fig. 3.

### Experiment 3

Taking the group (HG and LG) as the inter-subject factor and the task as the intra-subject factor (Experiment 2 and Experiment 3), two-way repeated-measures ANOVA was carried out to investigate the variation trend of task performance of the two groups in the process of increasing the experimental difficulty. In terms of the Go RT, the results revealed that the main effect of the group was significant (F_1,56_ = 5.72, *p* < 0.05, partial *η^2^* = 0.09); specifically, the Go RT of the HG was significantly shorter than that of the LG. In addition, the main effect of the task was significant (F_1,56_ = 281.43, *p* < 0.05, partial *η*^*2*^ = 0.83); specifically, the Go RT of Experiment 2 was significantly shorter than that of Experiment 3. Meanwhile, the interaction between the group and the task was not significant (F_1,56_ = 0.29, *p* > 0.05, partial *η^2^* = 0.005). Follow-up analyses revealed that the Go RT of the HG was significantly shorter than t.hat of the LG in Experiment 3.

In terms of the Go accuracy, the results revealed that the main effect of the group (F_1,56_ = 3.472, *p* > 0.05, partial *η*^*2*^ = 0.06) was not significant and the main effect of the task (F_1,56_ = 9.59, *p* < 0.05, partial *η*^*2*^ = 0.15) was significant. Moreover, the interaction between the group and the task was not significant (F_1,56_ = 2.31, *p* > 0.05, partial *η*^*2*^ = 0.04). Follow-up analyses indicated that the Go accuracy of LG significantly decreased from Experiment 2 to Experiment 3 (*p* < 0.05), while no significant change was observed for the HG (*p* > 0.05, Fig. 3). Meanwhile, the Go accuracy of the HG was significantly greater than that of the LG in Experiment 3 (*p* < 0.05).

**Figure 4:**
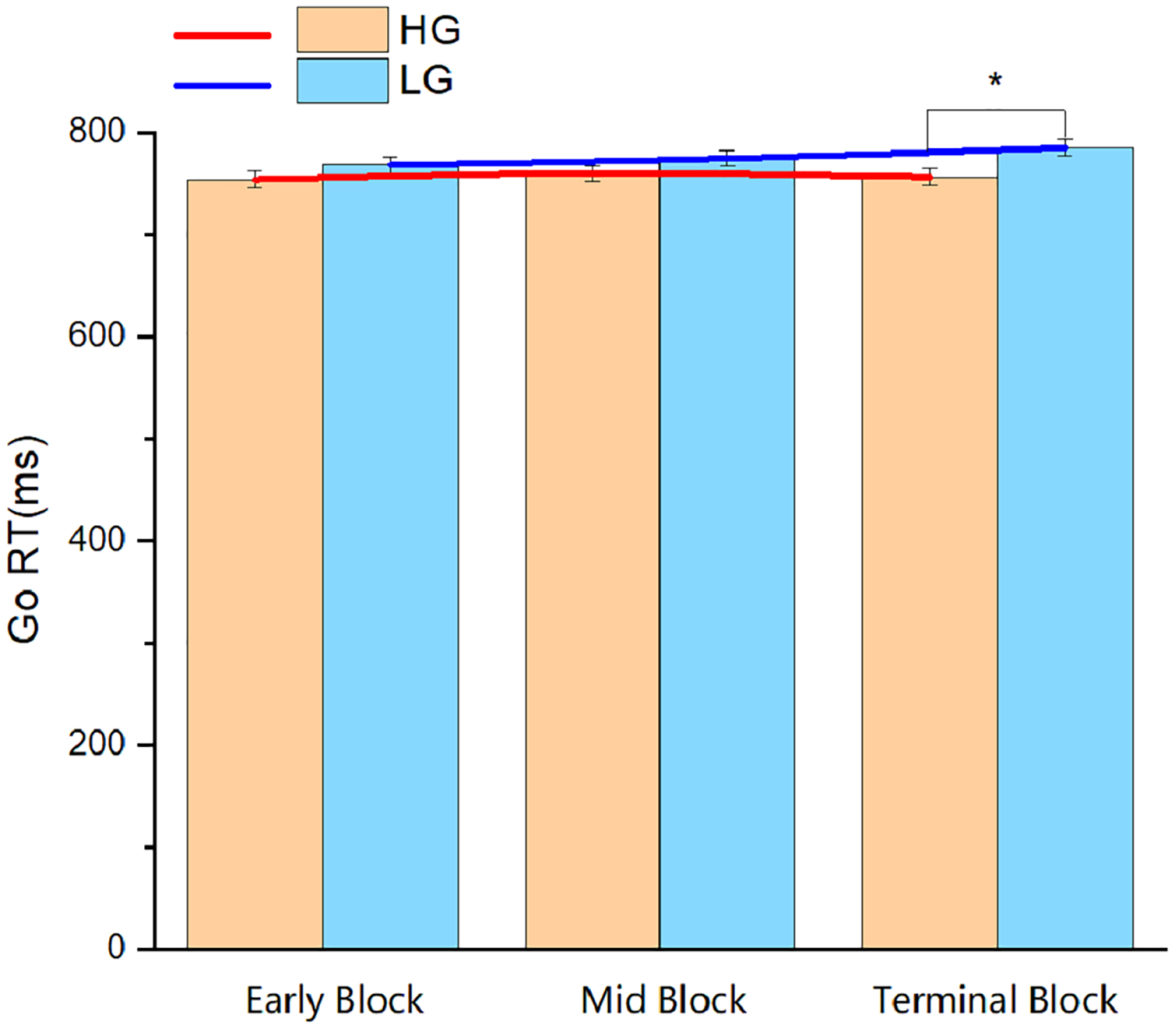
Reaction time of the go trials in the three blocks of Experiment 3. Abbreviations: Go RT, reaction time of the go trials; HG, higher physical activity group; LG, lower physical activity group. *p < 0.05.

**Figure 5:**
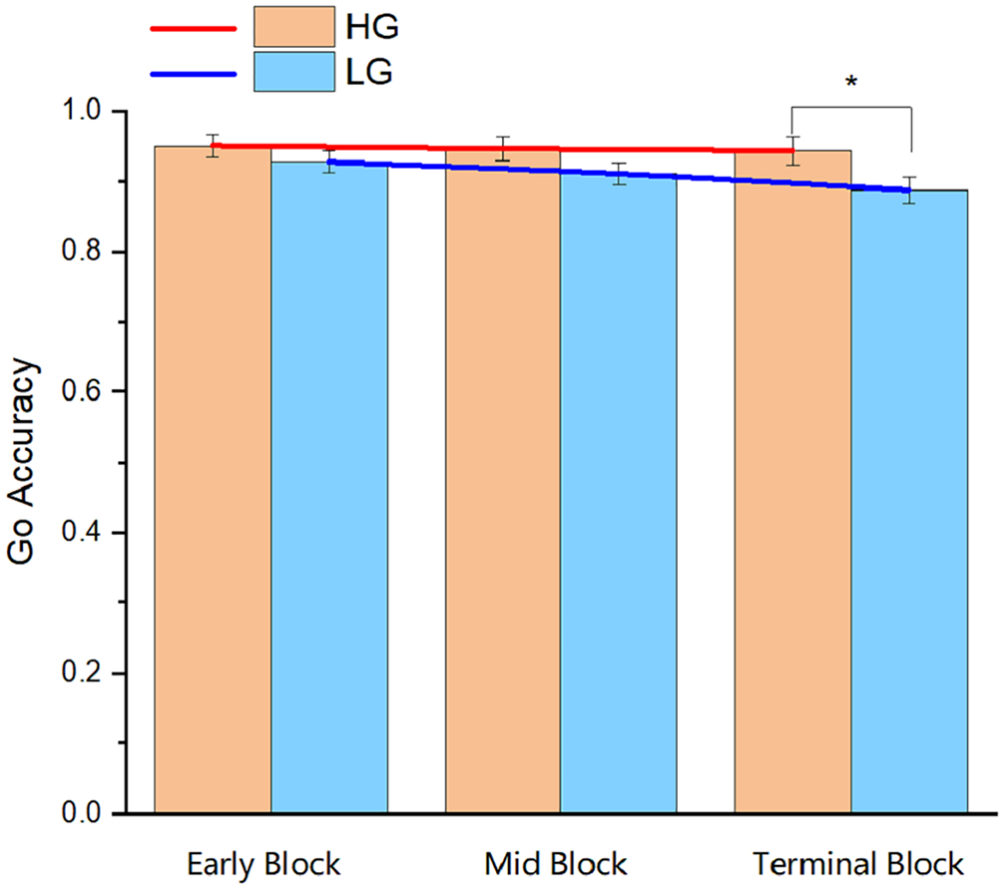
Accuracy of the go trials in the three blocks of Experiment 3. Abbreviations: HG, higher physical activity group; LG, lower physical activity group. *p < 0.05.

As the experiment consisted of three blocks, the attentional resources of individuals will be gradually lost with the progression of the task; therefore, it is of great significance to investigate the variation trend of the Go RT and Go accuracy in different blocks of the two groups. Repeated-measures ANOVA was conducted with the group (HG and LG) as the inter-subject factor and block (early block, mid-block, and terminal block) as the intra-subject factor. In terms of the Go RT, the results revealed that the main effect of the group was significant (F_1,56_ = 4.77, *p* < 0.05, partial *η*^*2*^ = 0.08). Meanwhile, the main effect of the block, and the interaction between the group and the block were not significant (*p* > 0.05). In addition, follow-up analyses revealed that the difference between the HG and the LG was significant in terminal block (Fig. 4). In terms of the Go accuracy, no significant results were found for the main effect of the block or the interaction between the group and the block (*p* > 0.05). However, a significant result was found for the main effect of the group (F_1,56_ = 4.54, *p* < 0.05, partial *η*^*2*^ = 0.08). Follow-up analyses indicated that the Go accuracy of the HG was significantly greater than that of the LG in terminal block (*p* < 0.05, Fig. 5).

## Discussion

In this study, the standard SST and two novel versions of the SST were used to investigate whether the association between PA and response inhibition stems from the fact that PA accelerates attentional capture of conflict signals, and further examined whether PA can regulate the allocation of attentional resources to better complete cognitively demanding task. The results of Experiment 1 showed that individuals with higher PA had a shorter SSRT, that is, PA was positively associated with response inhibition. Experiment 2 successfully separated the attentional capture in Experiment 1 to investigate whether the positive relationship between PA and response inhibition stems from the promotion of attentional capture brought by PA. The results revealed that individuals with higher PA had a shorter Go RT. The combination of Experiments 1 and 2 demonstrated that the improved response inhibition induced by attentional capture was associated with PA. Meanwhile, the results of Experiment 3 proved that, compared with individuals with lower PA, individuals with higher PA could efficiently allocate attentional resources to complete attentional capture. In general, this study provides a new perspective regarding the relationship between PA and response inhibition.

In Experiment 1, we found that the HG had a shorter SSRT than the LG, which is consistent with the findings of previous studies (Padilla et al., 2013; Chen & Muggleton, 2020). The underlying neural mechanism of this phenomenon can be explained by a study using event-related potential (ERP) (Lennox, Miller & Martin, 2019). P_3b_ is generally considered to reflect the allocation of attention resources during inhibitory processing (Polich, 2007). Once a stop trial was successfully inhibited in the SST, habitual exercisers showed a larger P_3b_ amplitude than non-exercisers. In addition, multimodal magnetic resonance imaging studies have provided evidence that the pre-SMA is typically responsible for inhibiting an ongoing movement (Sharp et al., 2010), and an empirical study has demonstrated that a 6-month exercise intervention can increase the grey matter volume in this region (Colcombe et al., 2006). In addition, the latest study found that increased amplitude of low-frequency fluctuation (ALFF) in right pre-SMA were observed in participants who received an 8-week exercise intervention (Huang et al., 2021). Therefore, we speculated that individuals who often participate in PA have a shorter SSRT, which may depend on the structural integrity and function of the pre-SMA. Our study did not find a significant difference between the HG and the LG in terms of the Go RT, which is also in agreement with the literature that PA facilitates the stop process instead of altering the go process (Padilla, Pérez & Andrés, 2014). In other words, PA seems to improve response inhibition and attentional performance.

Based on the results of Experiment 2, it was found that individuals in the HG showed a faster Go RT than those in the LG, indicating that PA can improve the performance of attentional capture. The current evidence suggests that rapid stimulus monitoring is a key determinant of psychophysical performance (Salinas & Stanford, 2013) and response inhibition is initiated by rIFG (Schaum et al., 2021). Therefore, combining the results of Experiments 1 and 2, we can infer that the positive relationship between PA and response inhibition stems from the promotion of attentional capture brought by PA. The corresponding electrophysiological evidence supports this view. The N_1_ component is considered as an important index related to visual attention, and current evidence indicates that an enhanced N_1_ amplitude can be observed in people with a higher PA level (Chang et al., 2013; Cheval et al., 2018). In the SST, a successful response in the stop trial can induce a significant increase in the N_1_ amplitude (Boehler et al., 2009), indicating that PA can enhance attentional capture in the early perceptual processing stage. Thus, PA affects response inhibition *via* attentional capture.

Comparison of the results of Experiments 2 and 3 revealed that the HG had a better performance than the LG in terms of the Go RT. As for the Go accuracy, there was no significant difference between the HG and the LG in Experiment 2; however, the accuracy of the LG decreased significantly from Experiment 2 to Experiment 3, which was not observed for the HG. Compared with Experiment 2, Experiment 3 not only required the subjects to respond to the signal but also to inhibit the signal, which demanded more attentional resources. Related studies have demonstrated that the cognitive advantage induced by PA can be reflected only when the test reaches a higher cognitive demand (Ishihara et al., 2020). Therefore, a significant difference in accuracy between the two groups was only observed in Experiment 3. In addition, Experiment 3 contained three blocks, and the subjects’ attentional resources were gradually consumed with the progression of the blocks. In terms of the Go RT, there was no significant difference between the two groups in the early or mid-blocks, but the difference between the two groups in the terminal block gradually increased. The results of Go accuracy were similar to those of the Go RT, and the difference between the HG and LG peaked in the terminal block. This finding is similar to the results of a classical experiment on working memory, that is, individuals with a high memory capacity did not have large fluctuations in contralateral delay activity with an increase of the difficulty level of the task, while the performance of individuals with a low memory capacity was the opposite (Vogel, McCollough & Machizawa, 2005). These results revealed that the LG was poor at maintaining task performance throughout all three blocks, which roughly led to a significant decline in the performance during the terminal block. In addition, no significant difference was found between the two groups in terms of the Go RT in the early and mid-blocks, which supports the idea that the cognitive benefits brought by PA are shown only when the task reaches the corresponding difficulty or intensity (Biddle & Asare, 2011). In addition, the findings of Experiment 3 can also be explained by the result of the latest meta-analysis (Hvid et al., 2021), which found that aerobic exercise training increased gray matter in inferior frontal gyrus, and Erika-Florence, Leech & Hampshire (2014) found that this region showed heightened activation during complex task, so we speculate that this may be the critical mechanism for the cognitive benefits brought by PA. It is worth noting that Tay et al. (2022) have revealed the interplay between inhibitory control and attentional capture. Their study found that P_D_ (a component of ERP associated with distractor suppression) but not N_2PC_ (a component of ERP associated with attentional orienting) was induced by a significant stimulus in no-go trials, indicating that inhibitory control prevented capture of visual attention (Tay et al., 2022). Different from our study in which the presence of red arrows related to attentional capture determines whether the subjects need to initiate inhibitory control, the paradigm used by Tay et al. (2022) determines whether the subjects need to engage in stimulus capture after inhibitory control is initiated. Therefore, future studies analysing the influence of PA on inhibitory control need to combine specific paradigms.

This study had some limitations that should be noted. First, this study had a cross-sectional experimental design, and the results need to be replicated with research using a longitudinal experimental design. Second, this study was an exploratory study, so it only compared the behavioural performances of the HG and the LG. Future research should further verify the results of this study by dividing the PA level more accurately with accelerometers. Finally, the paradigm involved in this study did not allow the measurement of proactive inhibition, and it is also crucial to investigate the mechanism of PA on proactive inhibition through the existing modified SST (Ikarashi et al., 2022).

## Conclusions

The results of this study indicate that PA is positively associated with response inhibition and that the positive relationship is associated with effective allocation of attentional resources for faster attentional capture. The findings of this study provide new insight for further research on the promotion of response inhibition by PA.

## Supplemental Information

10.7717/peerj.14083/supp-1 Supplemental Information 1 Raw data.

## Additional Information and Declarations

### Data Availability

The following information was supplied regarding data availability: The raw data is available in the Supplemental File.
